# Seneca Valley Virus Degrades STING via PERK and ATF6-Mediated Reticulophagy

**DOI:** 10.3390/v15112209

**Published:** 2023-11-03

**Authors:** Ling Bai, Rui Zhang, Haixue Zheng, Zhixiong Zhang, Zhidong Zhang, Yanmin Li

**Affiliations:** 1 State Key Laboratory of Veterinary Etiological Biology, Lanzhou Veterinary Research Institute, Chinese Academy of Agricultural Sciences, Lanzhou 730000, China; bai620ling@163.com (L.B.); haixuezheng@163.com (H.Z.); zxiongzhang@163.com (Z.Z.); 2 College of Animal Husbandry and Veterinary Medicine, Southwest Minzu University, Chengdu 610041, China; zhangrui.caas@foxmail.com

**Keywords:** SVV, STING, autophagy, UPR

## Abstract

Seneca Valley Virus (SVV), a member of the *Picornaviridae* family, is an emerging porcine virus that can cause vesicular disease in pigs. However, the immune evasion mechanism of SVV remains unclear, as does its interaction with other pathways. STING (Stimulator of interferon genes) is typically recognized as a critical factor in innate immune responses to DNA virus infection, but its role during SVV infection remains poorly understood. In the present study, we observed that STING was degraded in SVV-infected PK-15 cells, and SVV replication in the cells was affected when STING was knockdown or overexpressed. The STING degradation observed was blocked when the SVV-induced autophagy was inhibited by using autophagy inhibitors (Chloroquine, Bafilomycin A1) or knockdown of autophagy related gene 5 (ATG5), suggesting that SVV-induced autophagy is responsible for STING degradation. Furthermore, the STING degradation was inhibited when reticulophagy regulator 1 (FAM134B), a reticulophagy related receptor, was knocked down, indicating that SVV infection induces STING degradation via reticulophagy. Further study showed that in eukaryotic translation initiation factor 2 alpha kinase 3 (PERK)/activating transcription factor 6 (ATF6) deficient cells, SVV infection failed to induce reticulophagy-medaited STING degradation, indicating that SVV infection caused STING degradation via PERK/ATF6-mediated reticulophagy. Notably, blocking reticulophagy effectively hindered SVV replication. Overall, our study suggested that SVV infection resulted in STING degradation via PERK and ATF6-mediated reticulophagy, which may be an immune escape strategy of SVV. This finding improves the understanding of the intricate interplay between viruses and their hosts and provides a novel strategy for the development of novel antiviral drugs.

## 1. Introduction

Seneca Valley Virus (SVV) is a prototypic member of the *Senecavirus* genus within the *Picornaviridae* family and is a non-enveloped, positive-stranded RNA virus with a genome length of approximately 7.3 kb [1]. As an emerging porcine virus, SVV is able to cause vesicular disease in pigs, which is clinically indistinguishable from foot-and-mouth disease (FMD) and other swine vesicular diseases [2]. SVV infection has caused some economic consequences to the global swine industry, but there is no vaccine available for disease control [3]. Consequently, a better understanding of host–virus interaction contributes to identifying potential targets for the development of efficient antiviral drugs and vaccines.

STING (Stimulator of interferon genes), also known as MITA (Mediator of IRF3 activation) or MYPS (plasma membrane tetraspanner), is an evolutionarily conserved transmembrane protein that localizes to the ER (endoplasmic reticulum) membrane in immune and non-immune cells and has been identified as a key signaling molecule in innate immune responses [4,5]. STING is primarily activated by CDNs (cyclic dinucleotides) induced by invading pathogens (such as bacteria and viruses) [6,7,8]. After binding to CDNs, STING activates and interacts with TBK1 (TANK-binding kinase 1), which leads to relocation to the perinuclear regions of cells. Then, activated TBK1 phosphorylates the transcription factors IRF3 (interferon regulatory factor 3) and NF-κB (nuclear factor-κB), which translocates into the nucleus to innate immune gene transcription [9]. Thus, STING, as a key molecule associated with innate immune responses, is essential for controlling the activation of antiviral responses. Many DNA viruses and bacteria have been reported to activate STING-dependent innate immune responses, including HBV (Hepatitis B Virus), HSV (Herpes Simplex Virus), Mycobacterium tuberculosis, and Brucella abortus [10,11,12,13]. Moreover, a growing body of evidence suggests that STING is activated by the infection of various RNA viruses, such as SeV (Sendai virus), VSV (Vesicular Stomatitis Virus), NDV (Newcastle Disease Virus), JEV (Japanese Encephalitis Virus), and SARS-CoV-2 (Severe acute respiratory syndrome coronavirus 2) [14,15,16]. These observations suggest that STING-mediated antiviral responses restrict both DNA and RNA viruses. However, the virus has also evolved numerous strategies to suppress STING-induced innate immune signaling pathways. DNA virus infection induces interaction between MYSM1 (Myb-like, SWIRM, and MPN domains 1) and STING to suppress innate immune responses [17]. HPV18 (Human Papillomavirus Type 18) E7 combines with STING in a site-critical form for NF-κB activation to antagonize STING-triggered innate immune response activation [18]. However, little has been done to understand the mechanism by which SVV antagonizes STING-dependent antiviral responses.

Autophagy is an evolutionarily conserved lysosome-dependent degradation pathway in which intracellular contents such as proteins, organelles, and lipids are degraded to maintain cellular homeostasis [19]. Depending on the mechanisms for delivering cargo to lysosomes, autophagy is classified into three types: microautophagy, chaperone-mediated autophagy, and macroautophagy [20]. Macroautophagy is the best-characterized form of autophagy (hereafter referred to as autophagy), which involves the initiation of autophagy, the elongation and closure of the autophagic membrane, the maturation and fusion of autophagosomes with lysosomes, and the degradation and recycling of autophagosomes [21]. Selective autophagy, a major form of autophagy, targets the degradation of specific substrates, especially dysfunctional or superfluous organelles. Regarding different organelles, multiple types of organelle-specific autophagy have been reported, such as mitophagy (mitochondrion), ribophagy (ribosome), pexophagy (peroxisome), reticulophagy (endoplasmic reticulum, ER), lysophagy (lysosome), and nucleophagy (nuclear) [22]. The innate immune system activates autophagy to clear invading pathogens by the degradation and disposal of cytoplasmic components [23,24]. However, viruses have also evolved strategies to counteract the host’s antiviral response by exploiting the process of autophagy degradation. PEDV (Porcine epidemic diarrhea virus) induces autophagy to degrade the host antiviral protein HNRNPA1 (Heterogeneous nuclear ribonucleoprotein A1) through its N protein [25]. ASFV (African Swine Fever Virus) activates mitophagy to degrade TOMM70 (mitochondrial membrane 70) via SQSTM1 (Sequestosome 1)-related autophagosomes, resulting in inhibiting TOMM70-mediated innate immune response [26]. A previous study from our group also confirmed that FMDV (foot-and-mouth disease virus) utilizes autophagy to degrade STING [27].These studies further demonstrate that viruses use autophagy to degrade antiviral proteins through different strategies. To date, a few studies have shown that SVV-induced autophagy promotes the replication of SVV [28,29,30]. However, it is unclear whether the underlying mechanism of this process is related to STING.

Numerous studies revealed that both DNA viruses and RNA viruses are capable of activating autophagy via UPR (unfolded protein response) to promote viral replication [31,32,33]. For example, PRRSV (Porcine reproductive and respiratory syndrome virus) stimulated autophagy through the ER stress-dependent calcium pathway to promote its replication [34]. A previous study reported that SVV infection activated autophagy via EIF2AK3/PERK (eukaryotic translation initiation factor 2 alpha kinase 3) and ATF6 (activating transcription factor 6) of UPR [29]. However, the relationship between this process and the viral evasion from the host’s antiviral responses, particularly the STING-mediated antiviral responses, is still not fully understood.

In the present study, we demonstrated that SVV infection induces the degradation of STING through reticulophagy via the PERK/ATF6 of UPR, which would improve the understanding of SVV pathogenesis and host cellular antiviral mechanisms.

## 2. Materials and Methods

### 2.1. Cells and Viruses

Porcine kidney cells (PK-15 cells; ATCC, CCL-33), porcine Instituto Biologico-Rim Suino-2 cells (IBRS-2; ATCC, CL-101), and Lenti-X^TM^ 293T cells (Clontech, Mountain View, CA, USA, 632180) were cultured in Duldecco’s modified Eagle’s medium (DMEM; Gibco, Carlsbad, CA, USA, C14190500BT) supplemented with 10% FBS (Gibco, Carlsbad, CA, USA, 10099-141), 100 U/mL penicillin, and 50 μg/mL streptomycin (Gibco, Carlsbad, CA, USA, 15140-122) at 37 °C under 5% CO_2_ in a humidified incubator. The SVV strain (CH-FJ-2017) was isolated and stored in our laboratory [35]. SVV was propagated and virus titers were determined using Karber’s method and expressed as 50% tissue culture infective dose (TCID_50_) in IBRS-2 cells.

### 2.2. Antibodies and Reagents

The main antibodies used in this study were as follows: anti-STING antibody (Cell Signaling Technology, Boston, MA, USA, 50494S), anti-LC3B antibody (Cell Signaling Technology, Boston, MA, USA, 2775s), anti-β-tubulin antibody (Proteintech, Chicago, IL, USA, 66240-1-Ig), anti-PERK antibody (Proteintech, Chicago, IL, USA, 24390-1-AP), anti-FAM134B antibody (Proteintech, Chicago, IL, USA 21537-1-AP), anti-ATF6 antibody (Abcam, Cambridge, UK, ab122897), anti-ATG5 antibody (Novus Biological, Centennial, CO, USA, NB110-53818), anti-VP1 antibody (our laboratory), anti-3D antibody (our laboratory), anti-rabbit antibody (Bio-Rad, Berkeley, CA, USA, 170–6515), and anti-mouse antibody (Bio-Rad, Berkeley, CA, USA, 170–6516). The main reagents used in this study were as follows: MG132 (Merck & Co., Darmstadt, Germany, 474787), Z-VAD-FMK (Merck & Co., Darmstadt, Germany, 627610), CQ (Sigma, Darmstadt, Germany, 509272), Baf-A1 (LC Laboratories, Woburn, MA, USA, B-1080), and 4µ8C (Selleck, Houston, TX, USA, 14003–96-4).

### 2.3. Pharmaceutical Treatment and Virus Infection

Pharmaceutical treatment and virus infection were carried out as in previous studies [27,36]. Briefly, PK-15 cells were incubated for 24 h prior to infection with medium containing the following inhibitors, respectively: 50 μM Z-VAD-FMK, 20 μM MG132, 50 μM CQ, 50 μM Baf-A1, and 100 μM 4μ8C. Doses were calculated by the formula “Concentration (start) × Volume (start) = Concentration (final) × Volume (final)”. PK-15 cells were then inoculated by SVV (MOI = 1) and incubated at 37 °C for 1 h. Unbound viruses were removed by washing with pre-warmed PBS. The cells were cultured in DMEM medium supplemented with 2% FBS and the same concentration of these inhibitors as mentioned above in a humidified incubator (37 °C, 5% CO_2_).

### 2.4. Construction of Plasmids

STING cDNA (accession no. 3. NM_198282.4) was inserted into Plvx-puro lentiviral vectors to construct plasmid expressing 3 × Flag -tagged STING proteins. The primers for PCR are listed in the following:

STING Forward: 5′-CTAGCTCGAGATGCCCCACTCCAGCCTGCAT-3′

STING Reverse: 5′-CTAGGGATCCCTTGTACAGCTCGTCCATGCC-3′

For the construction of lentiviral shRNA vectors, shRNA was designed using BLOCK-iT™ RNAi Designer (Invitrogen, Carlsbad, CA, USA). The primers were synthesized and cloned into pLKO.1 vectors by digestion and ligation; shRNA sequences are shown in the following:

STING: 5′-GCTCGGATCCAAGCTTATAAT-3′

ATG5: 5′-GCACACCACTGAAATGGCATT-3′

FAM134B: 5′-GCGAAAGCTGGGAAGTTATCA-3′

PERK: 5′-GCAGATCACTAGTGATTATCA-3′

ATF6: 5′-GCTGTCCAATACACAGAAACC-3′

For the construction of lentiviral CRISPR-Cas9 vectors, sgRNA was designed using sgRNA Designer from Feng Zhang’s lab (Cambridge, MA, USA). The primers were synthesized and cloned into Lenti CRISPR v2 vectors by ligation; the sgRNA sequence is as follows:

STING: 5′-CCCCCAAAGGGCCACCAAGC-3′

### 2.5. Lentivirus Packing and Infection

The pLKO.1 plasmid, psPAX2 packaging plasmid, and pMD2.G envelope plasmid were mixed with the transfection reagent (Thermo Fisher Scientific, Carlsbad, CA, USA, L3000015) and added to 4 × 10^6^ Lenti-X™ 293 T cells. The cell culture was subjected to multiple freeze–thaw cycles, followed by filtration to obtain the supernatant containing the lentivirus. The supernatant was then stored at −80 °C as aliquots. PK-15 cells were infected with lentivirus supernatant containing 8 mg/mL polybrene for a 24-h period. Following this, fresh medium was added to the cells. After 24 h of infection, resistant cells were selected using either hygromycin (Invitrogen, Carlsbad, CA, USA, 10687010) or puromycin (Invitrogen, Carlsbad, CA, USA, A1113803).

### 2.6. Western Blot

Cells were incubated on ice with RIPA Lysis Buffer (Beyotime, Shanghai, China, P0013B) containing PMSF (Beyotime, Shanghai, China, ST506) for 5 min. The samples were denatured in Laemmli 2× concentration sample buffer (Sigma, Darmstadt, Germany, S3401) at 95 °C for 10 min. Then, the samples were separated on 10% acrylamide gel and the protein bands in the gel were transferred to the PVDF (polyvinylidene fluoride) membrane (Millipore, Darmstadt, Germany, ISEQ00010). The membranes were blocked in TBST (Solarbio, Beijing, China, T1085) containing (5%) skimmed milk powder (BD, Becton Drive Franklin Lakes, NJ, USA, 232100) at RT (room temperature) for 2 h and were then incubated with primary antibody dilution at 4 °C overnight. The membranes were washed three times with TBST for 10 min each time. Then, they were incubated with the corresponding species of secondary antibody dilution at RT for 2 h. After washing, membranes were visualized by enhanced chemiluminescence (Affinity, Liyang, China, 170–5061). Bands were detected using BLT GV6000 Plus or Bio-Rad ChemiDoc MP in automatic or manual exposure models. The results were exhibited as the density ratio between proteins and the load control (β-tubulin) by Image J software (NIH, 1.46r).

### 2.7. Quantitative Reverse Transcription Polymerase Chain Reaction

Cells were seeded in 35-mm dishes, and total RNA was isolated using a Gene JET^TM^ RNA Purification Kit (Thermo Fisher Scientific, Carlsbad, CA, USA, K0731) according to the manufacturer’s instruction. For cDNA synthesis, total RNA was reverse-transcribed using ReverTra Ace qPCR RT Master Mix with gDNA Remover (TOYOBO, OSAKA, Japan, FSQ-301). For the quantification of gene expression, SYBR green-based RT-qPCR was performed using SYBRTM Green Master Mix (Thermo, Carlsbad, CA, USA, A25741), and the thermal cycling conditions were set as per the manufacturer’s instructions. The primers used in qPCR are shown below:

GAPDH Forward: 5′-GTCGGTTGTGGATCTGACCT-3′

GAPDH Reverse: 5′-AGCTTGACGAAGTGGTCGTT-3′

STING Forward: 5′- CTCCCAGCAGATAGGACTGC-3′

STING Reverse: 5′-CCTTGTCTCGGATGGAGAAG-3′

SVV VP1 Forward: 5′-CACCGACAACGCCGAGAC-3′

SVV VP1 Reverse: 5′-GAGATCGGTCAAACAGGAATTTGAC-3′

### 2.8. Statistical Analysis

All experiments were repeated at least three times independently. The significance analysis was constructed with *t*-test or one-way ANOVA test using Graphpad Prism software, version 8.0; *p*-values were interpreted as follows: * *p* < 0.05; ** *p* < 0.01; *** *p* < 0.001.

## 3. Results

### 3.1. SVV Infection Reduced STING Protein Level and STING Inhibited SVV Replication

**Figure 1:**
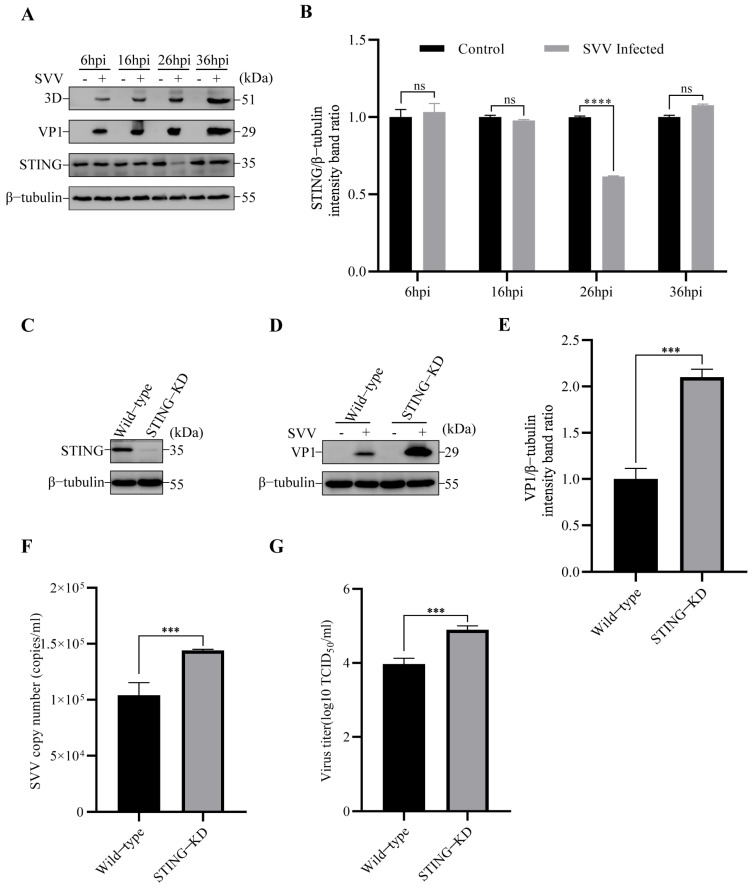
SVV infection reduced STING protein level, and SVV replication increased in STING-KD PK-15 cells. (**A**) PK-15 cells were infected or uninfected with SVV (MOI = 1) for 6 h, 16 h, 26 h, and 36 h. The levels of β-tubulin, STING (Stimulator of interferon genes), VP1, and 3D protein were detected by Western blot. (**B**) Intensity of STING and β-tubulin bands in (**A**) were analyzed by Image J, and the intensity ratio of STING/β-tubulin was shown (mean ± SD; *n* = 3; ns, no significance, **** *p* < 0.0001). (**C**) The cell lysates of wild-type and STING-KD PK-15 cells were collected. The levels of β-tubulin and STING protein were detected by Western blot. (**D**) Wild-type and STING-KD PK-15 cells were infected or uninfected with SVV (MOI = 1) for 26 h. The levels of β-tubulin and VP1 protein were detected by Western blot. (**E**) Intensity of VP1 and β-tubulin bands in (**D**) were analyzed by Image J, and the intensity ratio of VP1/β-tubulin was shown (mean ± SD; *n* = 3; *** *p* < 0.001). (**F**) Wild-type and STING-KD PK-15 cells were infected with SVV (MOI = 1, 26 hpi). The copy numbers of SVV in these cells were detected by qPCR (mean ± SD; *n* = 3; *** *p* < 0.001). (**G**) Wild-type and STING-KD PK-15 cells were infected with SVV (MOI = 1, 26 hpi). The virus titers of SVV in these cells were detected by TCID_50_ (mean ± SD; *n* = 3; *** *p* < 0.001).

To explore the effect of SVV infection on ER-anchored STING, we first detected the level of STING in SVV-infected PK-15 cells. The results by Western blot showed a significant reduction in the level of STING protein at 26 hpi (**** *p* < 0.0001), compared with control cells (Figure 1A,B). To investigate the role of STING in SVV infection, we constructed STING knockdown (KD) PK-15 cells (Figure 1C), and then the cells were infected with SVV (MOI = 1). We analyzed the effect of STING knockdown on SVV replication by Western blot, RT-qPCR, and TCID_50_. Compared to wild-type cells, the expression of SVV structural protein VP1 was found to increase in STING-KD cells (Figure 1D,E). Additionally, the results from copy numbers and viral titers indicated that SVV replication was enhanced in STING-KD cells (Figure 1F,G).

**Figure 2:**
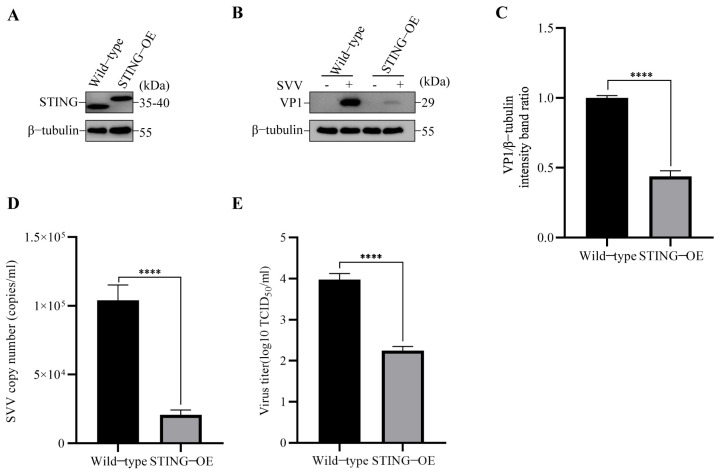
SVV replication was inhibited in STING-3×Flag OE PK-15 cells. (**A**) The cell lysates of wild-type and STING-3×Flag OE PK-15 cells were collected. The levels of β-tubulin and STING protein were detected by Western blot. (**B**) Wild-type and STING-3×Flag OE PK-15 cells were infected or uninfected with SVV (MOI = 1) for 26 h. The levels of β-tubulin and VP1 protein were detected by Western blot. (**C**) Intensity of VP1 and β-tubulin bands in (**B**) were analyzed by Image J, and the intensity ratio of VP1/β-tubulin was shown (mean ± SD; *n* = 3; **** *p* < 0.0001). (**D**) Wild-type and STING-3 × Flag OE PK-15 cells were infected with SVV (MOI = 1, 26 hpi). The copy numbers of SVV in these cells were detected by qPCR (mean ± SD; *n* = 3; **** *p* < 0.0001). (**E**) Wild-type and STING-3 × Flag OE PK-15 cells were infected with SVV (MOI = 1, 26 hpi). The virus titers of SVV in these cells were detected by TCID_50_ (mean ± SD; *n* = 3; **** *p* < 0.0001).

To further verify the role of STING in SVV infection, we also overexpressed STING with 3 × Flag (STING-OE) in PK-15 cells (Figure 2A), and then the cells were infected with SVV (MOI = 1). We analyzed the effect of STING overexpression on SVV replication using Western blot, RT-qPCR, and TCID_50_. As shown in Figure 2B,C, the level of VP1 in STING-OE cells was significantly lower than the level in wild-type cells. Moreover, the results of copy numbers and viral titers showed that SVV replication was inhibited in the STING-OE cells (Figure 2D,E). These results suggested that STING has an inhibitory impact on SVV infection in the cells.

### 3.2. SVV Infection Leads to the Degradation of STING via Autophagy

**Figure 3:**
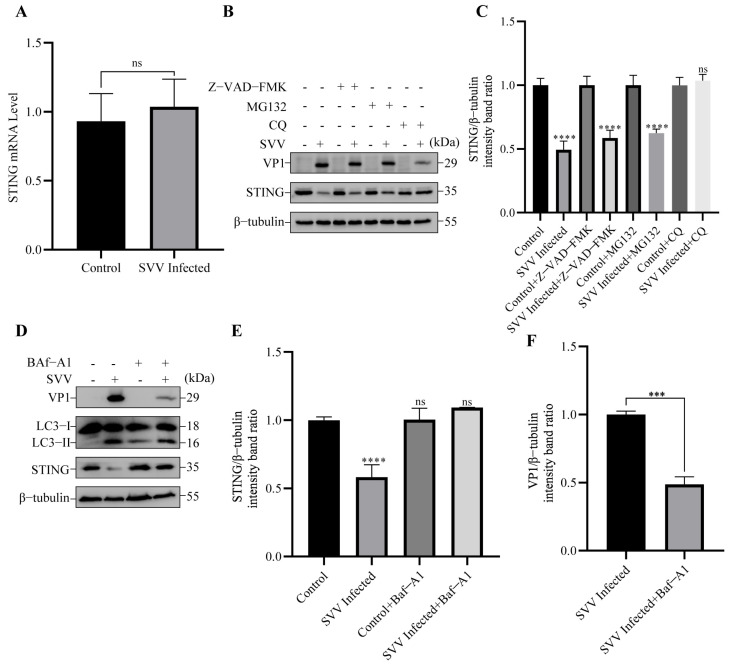
SVV-induced autophagy degraded STING. (**A**) PK-15 cells were infected or uninfected with SVV (MOI = 1) for 26 h. The relative expression of STING mRNA levels was measured by qPCR (mean ± SD; *n* = 3; ns, no significance). (**B**) PK-15 cells were infected or uninfected with SVV (MOI = 1) in the presence or absence of Z-VAD-FMK, MG-132, or chloroquine (CQ) for 26 h. The levels of β-tubulin, STING, and VP1 protein were detected by Western blot. (**C**) Intensity of STING and β-tubulin bands in (**B**) were analyzed by Image J, and the intensity ratio of STING/β-tubulin was shown (mean ± SD; *n* = 3; ns, no significance, **** *p* < 0.0001). (**D**) PK-15 cells were infected or uninfected with SVV (MOI = 1) in the presence or absence of bafilomycin A1 (Baf-A1) for 26 h. The levels of β-tubulin, STING, microtubule associated protein 1 light chain 3 beta (LC3), and VP1 protein were detected by Western blot. (**E**) Intensity of STING and β-tubulin bands in (**D**) were analyzed by Image J, and the intensity ratio of STING/β-tubulin was shown (mean ± SD; *n* = 3; ns, no significance, **** *p* < 0.0001). (**F**) Intensity of VP1 and β-tubulin bands in (**D**) were analyzed by Image J, and the intensity ratio of VP1/β-tubulin was shown (mean ± SD; *n* = 3; *** *p* < 0.001).

To investigate the reason for the reduction of STING, we first detected the mRNA level of STING in SVV-infected PK-15 cells at 26 hpi. As shown in Figure 3A, there was no difference in the mRNA level of STING between SVV-infected and uninfected cells. Thus, the reduction of STING was not attributed to SVV-mediated transcriptional inhibition. To further identify the pathway responsible for STING degradation, we pre-treated cells with caspase inhibitor Z-VAD-FMK, proteasome inhibitor MG132, and autophagy inhibitor CQ (chloroquine), respectively. Subsequently, the level of STING in these cells was detected using Western blot. As shown in Figure 3B,C, compared to control cells, treatment with Z-VAD-FMK and MG132 failed to block SVV-induced STING degradation; in contrast, CQ successfully prevented STING reduction in PK-15 cells infected with SVV. Furthermore, treatment with Baf-A1 (bafilomycin A1), another inhibitor of autophagy, also inhibited the degradation of STING in SVV-infected PK-15 cells (Figure 3D,E). Importantly, the inhibition of autophagy by Baf-A1 blocked the expression of the SVV structural VP1 protein (Figure 3D,F).

**Figure 4:**
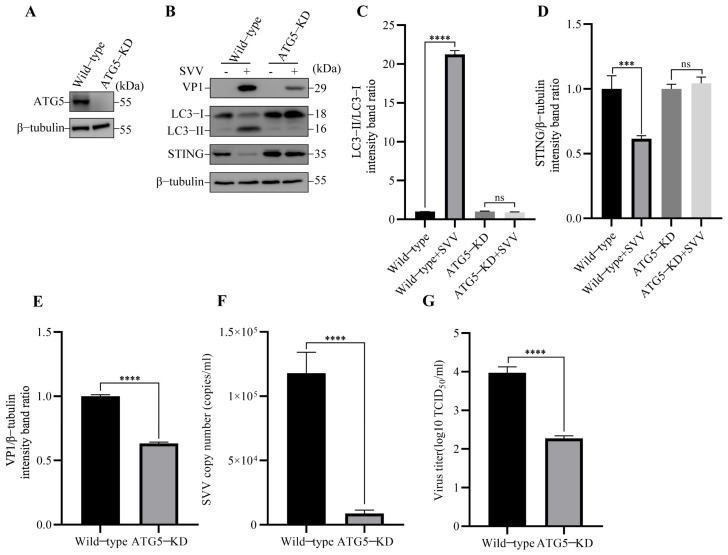
SVV-induced autophagy degraded STING via ATG5. (**A**) The cell lysates of wild-type and autophagy related gene 5 (ATG5)-KD PK-15 cells were collected. The levels of β-tubulin and ATG5 protein were detected by Western blot. (**B**) Wild-type and ATG5-KD PK-15 cells were infected or uninfected with SVV (MOI = 1) for 26 h. The levels of β-tubulin, STING, LC3, and VP1 protein were detected by Western blot. (**C**) Intensity of LC3-I and LC3-II bands in (**B**) were analyzed by Image J, and the intensity ratio of LC3-II/LC3-I was shown (mean ± SD; *n* = 3; ns, no significance, **** *p* < 0.0001). (**D**) Intensity of STING and β-tubulin bands in (**B**) were analyzed by Image J, and the intensity ratio of STING/β-tubulin was shown (mean ± SD; *n* = 3; ns, no significance, *** *p* < 0.001). (**E**) Intensity of VP1 and β-tubulin bands in (**B**) were analyzed by Image J, and the intensity ratio of VP1/β-tubulin was shown (mean ± SD; *n* = 3; **** *p* < 0.0001). (**F**) Wild-type and ATG5-KD PK-15 cells were infected with SVV (MOI = 1, 26 hpi). The copy numbers of SVV in these cells were detected by qPCR (mean ± SD; *n* = 3; **** *p* < 0.0001). (**G**) Wild-type and ATG5-KD PK-15 cells were infected with SVV (MOI = 1, 26 hpi). The virus titers of SVV in these cells were detected by TCID_50_ (mean ± SD; *n* = 3; **** *p* < 0.0001).

To further confirm the role of autophagy induction in degrading STING, the autophagy related gene 5 (ATG5) knockdown (KD) PK-15 cells were constructed (Figure 4A), which is the key component in the formation of autophagosomes [20], and then the cells were infected with SVV. Compared to wild-type cells, SVV infection was unable to induce autophagy in ATG5-KD cells (Figure 4B,C). Importantly, the knockdown of ATG5 was able to prevent STING degradation in SVV-infected ATG5-KD cells (Figure 4B,D). To further investigate the connection between autophagy and SVV replication, we examined the effect of ATG5 knockdown on SVV replication by Western blot, RT-qPCR, and TCID_50_. As shown in Figure 4B,E, the protein level of SVV VP1 was significantly lower in ATG5-KD cells compared to wild-type cells. We also measured SVV copy numbers and viral titers using RT-qPCR and TCID_50_, and the results indicated that SVV replication was inhibited in ATG5-KD cells compared to wild-type cells (Figure 4F,G). These results indicated that the induction of autophagy through ATG5 is responsible for STING degradation and SVV replication. Taken together, these results suggest that SVV-induced degradation of STING is associated with the activation of autophagy.

### 3.3. SVV-Induced Reticulophagy Degrades STING via PERK and ATF6

**Figure 5:**
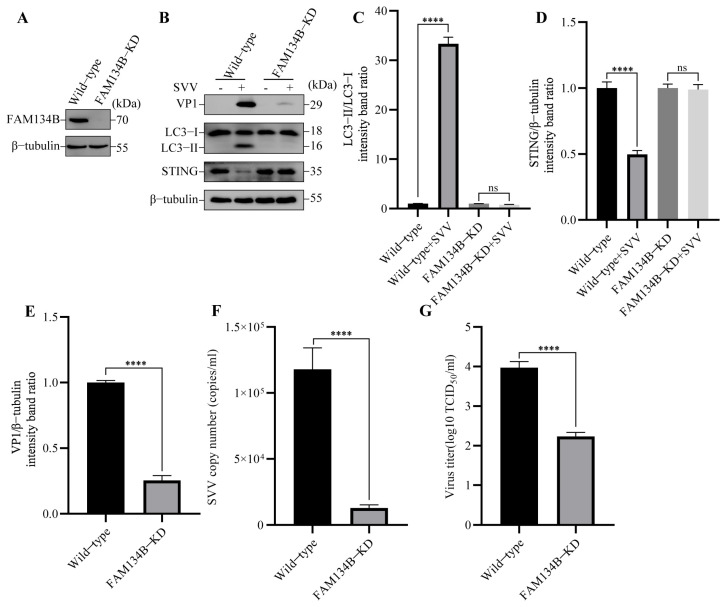
SVV degraded STING via reticulophagy. (**A**) The cell lysates of wild-type and reticulophagy regulator 1 (FAM134B)-KD PK-15 cells were collected. The levels of β-tubulin and FAM134B protein were detected by Western blot. (**B**) Wild-type and FAM134B-KD PK-15 cells were infected or uninfected with SVV (MOI = 1) for 26 h. The levels of β-tubulin, STING, LC3, and VP1 protein were detected by Western blot. (**C**) Intensity of LC3-I and LC3-II bands in (**B**) were analyzed by Image J, and the intensity ratio of LC3-II/LC3-I was shown (mean ± SD; *n* = 3; ns, no significance, **** *p* < 0.0001). (**D**) Intensity of STING and β-tubulin bands in (**B**) were analyzed by Image J, and the intensity ratio of STING/β-tubulin was shown (mean ± SD; *n* = 3; ns, no significance, **** *p* < 0.0001). (**E**) Intensity of VP1 and β-tubulin bands in (**B**) were analyzed by Image J, and the intensity ratio of VP1/β-tubulin was shown (mean ± SD; *n* = 3; **** *p* < 0.0001). (**F**) Wild-type and FAM134B-KD PK-15 cells were infected with SVV (MOI = 1, 26 hpi). The copy numbers of SVV in these cells were detected by qPCR (mean ± SD; *n* = 3; **** *p* < 0.0001). (**G**) Wild-type and FAM134B-KD PK-15 cells were infected with SVV (MOI = 1, 26 hpi). The virus titers of SVV in these cells were detected by TCID_50_ (mean ± SD; *n* = 3; **** *p* < 0.0001).

Reticulophagy (also known as ER-phagy) is a selective form of autophagy that protects cells from damage caused by excessive ER stress. Considering that STING is located in the ER membrane and SVV infection activates autophagy via ER stress [29], we speculate that the SVV infection induced by the degradation of STING might be due to selective organelle-specific reticulophagy. To further verify which type of autophagy (pan-autophagy or reticulophagy) is activated by SVV infection, we knocked down the ER-resident receptor FAM134B/RETREG1 (reticulophagy regulator 1) (Figure 5A), which interacts with the autophagy modifier MAP1LC3B/LC3 (microtubule associated protein 1 light chain 3 beta) and is engaged in reticulophagy but not pan-autophagy [22]. In FAM134B-KD PK-15 cells, SVV-triggered autophagy and STING degradation were blocked (Figure 5B–D). Additionally, we analyzed the effect of FAM134B knockdown on SVV replication by Western blot, RT-qPCR, and TCID_50_. SVV VP1 expression (Figure 5B,E), copy numbers (Figure 5F), and virus titers (Figure 5G) were inhibited compared to wild-type cells. These data suggested that SVV infection induces reticulophagy to degrade STING.

**Figure 6:**
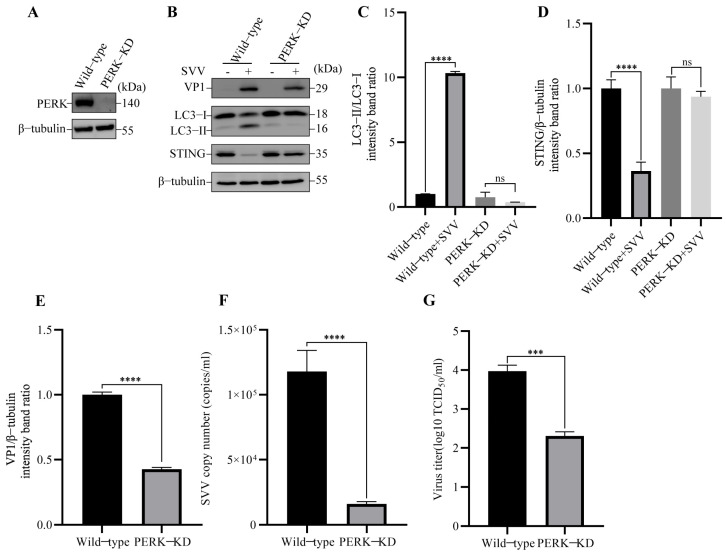
SVV induced reticulophagy via PERK to degrade STING. (**A**) The cell lysates of wild-type and eukaryotic translation initiation factor 2 alpha kinase 3 (PERK)-KD PK-15 cells were collected. The levels of β-tubulin and PERK protein were detected by Western blot. (**B**) Wild-type and PERK-KD PK-15 cells were infected or uninfected with SVV (MOI = 1) for 26 h. The levels of β-tubulin, STING, LC3, and VP1 protein were detected by Western blot. (**C**) Intensity of LC3-I and LC3-II bands in (**B**) were analyzed by Image J, and the intensity ratio of LC3-II/LC3-I was shown (mean ± SD; *n* = 3; ns, no significance, **** *p* < 0.0001). (**D**) Intensity of STING and β-tubulin bands in (**B**) were analyzed by Image J, and the intensity ratio of STING/β-tubulin was shown (mean ± SD; *n* = 3; ns, no significance, **** *p* < 0.0001). (**E**) Intensity of VP1 and β-tubulin bands in (**B**) were analyzed by Image J, and the intensity ratio of VP1/β-tubulin was shown (mean ± SD; *n* = 3; **** *p* < 0.0001). (**F**) Wild-type and PERK-KD PK-15 cells were infected with SVV (MOI = 1, 26 hpi). The copy numbers of SVV in these cells were detected by qPCR (mean ± SD; *n* = 3; **** *p* < 0.0001). (**G**) Wild-type and PERK-KD PK-15 cells were infected with SVV (MOI = 1, 26 hpi). The virus titers of SVV in these cells were detected by TCID_50_ (mean ± SD; *n* = 3; *** *p* < 0.001).

Previous studies have shown that SVV induced autophagy via PERK and ATF6 of the UPR [29], and our previous results suggested that SVV-induced reticulophagy is responsible for STING degradation. Thus, we hypothesized that the degradation of STING might be induced by SVV-triggered reticulophagy via UPR. The UPR is initiated by three major sensors (PERK, ERN1/IRE1α (endoplasmic reticulum to nucleus signaling 1), and ATF6). We wondered whether the three sensors are all required for reticulophagy-mediated STING degradation. We first constructed PERK knockdown (KD) cells (Figure 6A). In PERK-KD cells, the SVV-induced autophagy and STING degradation were inhibited (Figure 6B–D). Furthermore, we also analyzed the effect of PERK knockdown on SVV replication by Western blot, RT-qPCR, and TCID_50_. As shown in Figure 6B,E–G, SVV VP1 expression, copy numbers, and virus titers were inhibited compared with wild-type cells. These results suggested that PERK plays a significant role in SVV-induced reticulophagy-mediated STING degradation.

**Figure 7:**
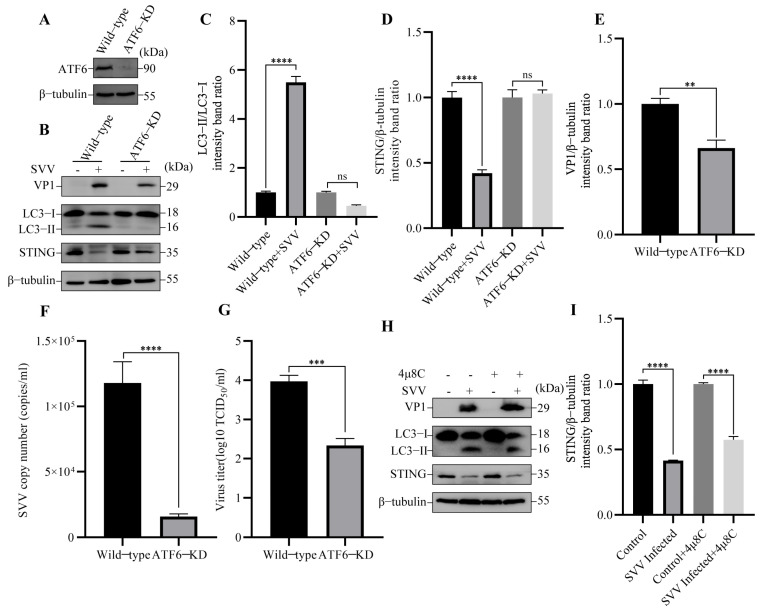
SVV induced reticulophagy via ATF6 to degrade STING. (**A**) The cell lysates of wild-type and activating transcription factor 6 (ATF6)-KD PK-15 cells were collected. The levels of β-tubulin and ATF6 protein were detected by Western blot. (**B**) Wild-type and ATF6-KD PK-15 cells were infected or uninfected with SVV (MOI = 1) for 26 h. The levels of β-tubulin, STING, LC3, and VP1 protein were detected by Western blot. (**C**) Intensity of LC3-I and LC3-II bands in (**B**) were analyzed by Image J, and the intensity ratio of LC3-II/LC3-I was shown (mean ± SD; *n* = 3; ns, no significance, **** *p* < 0.0001). (**D**) Intensity of STING and β-tubulin bands in (**B**) were analyzed by Image J, and the intensity ratio of STING/β-tubulin was shown (mean ± SD; *n* = 3; ns, no significance, **** *p* < 0.0001). (**E**) Intensity of VP1 and β-tubulin bands in (**B**) were analyzed by Image J, and the intensity ratio of VP1/β-tubulin was shown (mean ± SD; *n* = 3; ** *p* < 0.01). (**F**) Wild-type and ATF6-KD PK-15 cells were infected with SVV (MOI = 1, 26 hpi). The copy numbers of SVV in these cells were detected by qPCR (mean ± SD; *n* = 3; **** *p* < 0.0001). (**G**) Wild-type and ATF6-KD PK-15 cells were infected with SVV (MOI = 1, 26 hpi). The virus titers of SVV in these cells were detected by TCID_50_ (mean ± SD; *n* = 3; *** *p* < 0.001). (**H**) PK-15 cells were infected or uninfected with SVV (MOI = 1) in the presence or absence of 4μ8C for 26 h. The levels of β-tubulin, STING, LC3, and VP1 protein were detected by Western blot. (**I**) Intensity of STING and β-tubulin bands in (**H**) were analyzed by Image J, and the intensity ratio of STING/β-tubulin was shown (mean ± SD; *n* = 3; **** *p* < 0.0001).

To determine whether other UPR sensors are necessary for the induction of reticulophagy and the decline of STING protein induced by SVV, we knocked down the expression of ATF6 in PK-15 cells (Figure 7A). In ATF6-KD PK-15 cells, SVV-induced reticulophagy and STING degradation were hindered (Figure 7B–D) compared with wild-type cells. These results indicated that ATF6 also plays an indispensable role in SVV-triggered reticulophagy and STING degradation. Additionally, we also compared SVV replication between ATF6-KD and wild-type cells by Western blot, RT-qPCR, and TCID_50_. As shown in Figure 7B,E–G, SVV replication was inhibited in ATF6-KD cells. To dissect the impact of IRE1α-XBP1 at the molecular level, we used IRE1α-specific inhibitor 4μ8C to restrict the IRE1α-XBP1 pathway. As shown in Figure 7H,I, 4μ8C had no effect on SVV-induced reticulophagy-mediated STING degradation in PK-15 cells compared with the control cells. Our findings indicated that the PERK and ATF6 sensors of UPR are crucial for reticulophagy-mediated STING degradation induced by SVV.

## 4. Discussion

Innate immune response is the first line of host defense against invading pathogens. It is widely believed that STING’s antiviral responses to abnormal DNA from pathogens or cytoplasm induces the type I Interferon (IFN-I) pathway [37,38,39]. Recently, a growing body of evidence has proven that STING can also transmit signals from RNA viruses to activate IFN-I response [40,41,42]. During infection with SeV and VSV, the deficiency of STING blocks the activation of IRF3 and the expression of ISG and IFN-β. Thus, STING-KD cells are more sensitive to these viruses [43]. Here, our study also observed an increase in SVV replication in STING-KD PK-15 cells, while SVV replication was inhibited in STING-OE PK-15 cells. These results suggest that STING has an inhibitory effect on SVV replication. Similar to our results, the overexpression of STING in TB1 Lu (T. brasiliensis 1 lung) cells significantly inhibited VSV replication. In contrast, the IFN-β response triggered by VSV was severely hindered in STING-KD cells, and VSV replication was increased [44]. In addition to the interferon pathway, STING can also inhibit RNA virus infection by inhibiting global translation and by triggering cell apoptosis [45,46]. Regardless of the specific mechanism, it is universally observed that STING inhibits RNA virus replication. Our research further supports this point, but the specific mechanism of STING during SVV infection remains to be confirmed, and will be in our subsequent studies.

Autophagy is a physiological process that maintains host health by degrading cellular components through autophagosomes. As a defense strategy of the host, autophagy can be activated by viruses at any step of the viral life cycle. Accumulating evidence indicates that various viruses have developed their special strategies to hijack autophagy [47,48,49,50]. It has been investigated that viruses can manipulate the process of autophagy to degrade molecules of innate immunity, such as TBK1, cGAS (Cyclic GMP-AMP Synthase), and MAVS (Mitochondrial Antiviral Signaling Protein), in order to achieve their goal of survival and propagation [51,52,53]. Moreover, both DNA and RNA viruses have evolved strategies to exploit autophagy as a means to evade the antiviral function of STING. For example, ASFV promoted the autophagic degradation of STING, resulting in negative regulation of the cGAS-STING-mediated IFN-I pathway [54,55]. In addition to DNA viruses, RNA viruses also target STING to antagonize its antiviral response. SVCV (Spring Viremia of Carp Virus) downregulated cellular IFN production by utilizing the autophagolysosomal-dependent pathway to degrade STING [56]. In this study, we discovered that SVV infection could degrade STING via autophagy. These data are similar to our previous publication [27], which demonstrated that STING is degraded by autophagy during infection with FMDV, another picornavirus that infects pigs. However, a previous study suggested that SVV cannot reduce STING protein at 0, 8, and 16 hpi [57], which might be due to different hours post infection with SVV, as compared to our study (26 hpi). Overall, our study provides further evidence that reducing STING is a common strategy of DNA and RNA viruses.

There have been numerous investigations reported on the relationship between SVV infection and autophagy. SVV infection activates autophagy to promote viral infection in pig cells [28]. Thus, the inhibition of autophagy by the knockdown of ATG7 (autophagy related gene 7) inhibits SVV replication [29,57]. Consistent with this, our study also revealed that inhibiting autophagy by the knockdown of ATG5 and inhibitors (CQ and Baf-A1) effectively suppresses viral replication, providing further evidence for the role of autophagy in promoting SVV replication. Similar phenomena have also been found in other picornaviruses. Inhibition of autophagy (late or early stage) by various inhibitors all inhibited EV-A71 (Enterovirus 71) replication [58,59]. Similar to EV-71, the inhibition of autophagy with inhibitors and the knockdown of ATG7 was shown to suppress CVB3 (Coxsackievirus B3) and poliovirus replication [60,61]. FMDV replication is suppressed due to the inhibition of autophagy by either 3-MA or by small RNA interference (LC3 or autophagy related gene 12) [62]. EV-D68 (Enterovirus 68) exploits induced autophagy for its own benefit, and the viral yield is compromised when autophagy is inhibited by depleting ATG7 or treatment with Baf-A1 [63]. It is worth noting that, apart from picornaviruses, such biological phenomena have also been observed in PRV (Pseudorabies virus) and FeHV-1 (Feline herpesvirus 1) of the *Herpesviridae* family. It was found that induction of autophagy with rapamycin could promote FeHV-1 and PRV replication, while inhibition of autophagy with Baf-A1, CQ, or 3-MA (3-Methyladenine) inhibits viral replication, suggesting that autophagy promotes PRV and FeHV-1 replication [64,65]. In turn, viral infection can also regulate autophagy. It has been found that FeHV-1 and PRV infection regulate autophagy via PI3K/Akt/mTOR or Wnt/β-catenin, respectively [66,67]. Likewise, SVV can also activate the AKT-AMPK-MAPK-MTOR-dependent autophagy signaling pathway with its VP1, VP3, and 3C proteins [30]. Additionally, SVV also activates autophagy to decrease the expression of antiviral proteins such as cGAS [57]. Our study has found that SVV can also utilize the autophagy pathway to degrade another antiviral protein, STING. These findings suggest that autophagy plays a crucial role in the SVV life cycle in the cells.

Reticulophagy is one of the major forms of organelle-specific autophagy, targeting specific cargoes of ER cisternae filled with faulty proteins and lipids for degradation [68]. Upon stimulation, ER-localized sensors, including PERK, IRE1α, and ATF6, can detect the alterations of the ER environment and subsequently initiate several mechanisms, including autophagy [69]. For instance, autophagy is induced by HCV (Hepatitis C virus) through PERK/ATF6 of the UPR pathway [70]. SADS-CoV (Swine acute diarrhea syndrome coronavirus) activated autophagy through IRE1α of the UPR pathway [71]. Furthermore, inhibiting UPR not only hampers autophagy but also impedes viral replication during SADS-CoV and PEDV infection [71,72]. Although different viruses may employ different UPR receptors, virus-induced autophagy via UPR appears to be a common strategy. This phenomenon has also been confirmed during SVV infection [29], in which SVV activates autophagy through the PERK/ATF6 pathway. Our study further demonstrates that SVV infection induces STING degradation via PERK/ATF6-mediated reticulophagy. Consistently, our previous study also determined that the other picornavirus, FMDV, degrades STING through PERK-mediated reticulophagy [27]. These results indicate a possibility that STING degradation via virus-induced reticulophagy may serve as an immune escape strategy for SVV to replicate in the cells. However, further study is required to address this point.

## 5. Conclusions

**Figure 8:**
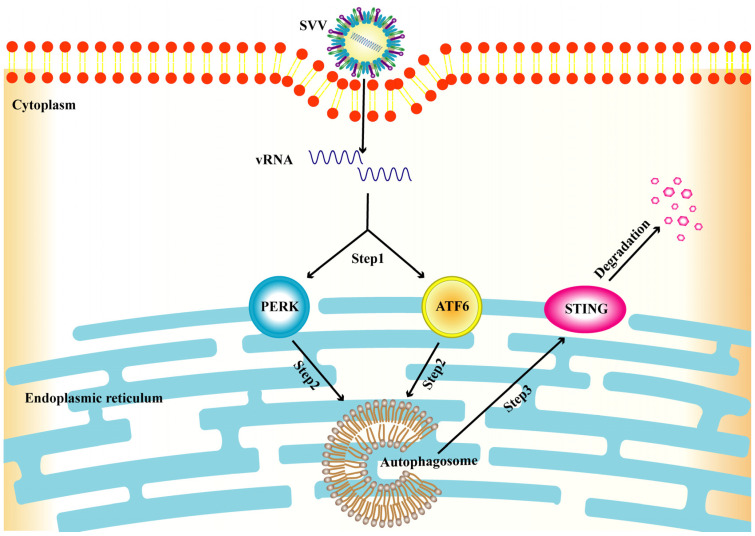
A model of SVV-induced STING degradation via PERK and ATF6-mediated reticulophagy. Step 1, SVV induces UPR via PERK and ATF6. Step 2, activation of PERK and ATF6 leads to reticulophagy. Step 3, STING is finally degraded through reticulophagy.

In summary, this study demonstrated that SVV infection activates reticulophagy via PERK and the ATF6 of UPR to degrade STING (Figure 8) and also substantiated the link between PERK/ATF6-mediated reticulophagy and STING degradation. Furthermore, the inhibition of reticulophagy reduced SVV replication. Further study is required to understand the mechanism of the degradation of STING via the autophagy involved in SVV replication.

## Data Availability

Original data files are available on request.
